# Thiazide-associated hyponatremia attenuates the fracture-protective effect of thiazide: A population-based study

**DOI:** 10.1371/journal.pone.0208712

**Published:** 2018-12-07

**Authors:** Lii-Jia Yang, Ping-Hsun Wu, Teng-Hui Huang, Ming-Yen Lin, Jer-Chia Tsai

**Affiliations:** 1 Division of Nephrology, Department of Internal Medicine, Kaohsiung Medical University Hospital, Kaohsiung, Taiwan; 2 Institute of Clinical Medicine, College of Medicine, Kaohsiung Medical University, Kaohsiung, Taiwan; 3 Faculty of Renal Care, College of Medicine, Kaohsiung Medical University, Kaohsiung, Taiwan; 4 Master of Public Health Degree Program, College of Public Health, National Taiwan University, Taipei, Taiwan; Medical College of Wisconsin, UNITED STATES

## Abstract

**Background:**

Thiazide, a first-line therapy for hypertension, lowers blood pressure, increases bone mineral density, and reduces the risk of fractures. However, hyponatremia, an adverse effect of thiazide, is associated with increased risk of osteoporosis and fractures. It is currently unclear whether thiazide-associated hyponatremia (TAH) outweighs the protective effects of thiazide.

**Methods:**

Using data from Taiwan’s National Health Insurance Research Database, we identified patients who were prescribed thiazide between 1998 and 2010. Those diagnosed with hyponatremia within three years after initiation of thiazide were selected for the TAH group. Thiazide users without hyponatremia were selected for the control group. The association between TAH and fracture risk was further evaluated using multivariable Cox regression models adjusted for comorbidities and medications. Subjects were followed up from the index date until the appearance of a fracture, death, or the end of a 3-year period.

**Results:**

A total of 1212 patients were included in the TAH group, matched with 4848 patients in the control group. The incidence rate of fracture was higher in the TAH group than in the control group (31.4 versus 20.6 per 1000 person-years). TAH was associated with a higher risk of total fractures (adjusted hazard ratio [aHR]: 1.47, 95% confidence interval [CI] = 1.15–1.88), vertebra fractures (aHR: 1.84, 95% CI = 1.12–3.01), and hip fractures (aHR: 1.66, 95% CI = 1.12–2.46) after controlling for comorbidities and other medications.

**Conclusions:**

Thiazide users with hyponatremia have a higher risk of fracture than thiazide users without hyponatremia. The fracture-protective effect of thiazide is attenuated by TAH.

## Introduction

Thiazide, a widely-used diuretic, is recommended as one of the first-line therapies for patients with hypertension [1]. In addition to lowering blood pressure, thiazide increases bone mineral density (BMD) [2], and reduces the risk of fracture [3].

Thiazide increases BMD via several mechanisms. First, thiazide induces positive calcium balance by decreasing renal calcium excretion [4]. During volume depletion, thiazide increases proximal sodium and water reabsorption, which leads to increased passive proximal calcium reabsorption [5]. Without volume depletion, thiazide increases calcium reabsorption in distal convoluted tubules by upregulating apical calcium channel transient receptor potential vanilloid 5 [6]. Second, thiazide exerts a direct osteoanabolic effect by stimulating osteoblast differentiation [7,8] and inhibits osteoclastic bone resorption by inhibiting carbonic anhydrase [9]. A Cochrane meta-analysis showed that thiazide use is associated with a 24% reduction in hip fracture risk, though it is uncertain whether this beneficial effect comes from increased BMD [3]. The effect of increasing BMD [10,11] and reducing fracture risk [12,13] seems to be linked to the duration of thiazide use.

However, thiazide possesses adverse effects. Thiazide exposure has been shown to be associated with substantially higher risk of hyponatremia [14], especially in patients with certain risk factors, such as elderly patients, female patients [15], and patients with low body weight [16]. Hyponatremia, though usually benign and asymptomatic, is significantly associated with increased risk of osteoporosis [17]. In animal models, hyponatremia has been shown to stimulate osteoclast and resorptive activity via induction of oxidative stress [18] and elevated circulating arginine-vasopressin [19]. Hyponatremia is also significantly associated with a higher risk of fracture [20]. In elderly patients with fragility fractures, hyponatremia has been reported highly prevalent (26%) [21]. Even mild chronic hyponatremia (Na 132 ± 5 mmol/L) has been associated with greatly increased risk of hip fracture in elderly patients [22]. Hyponatremia-induced osteoporosis and bone fragility may increase fracture risk [23]. Chronic hyponatremia has a significant adverse impact on cognitive function and gait stability and is associated with increased risk of falls, which could be another cause of hyponatremia-related fractures [24].

It is currently not clear whether the adverse effects associated with hyponatremia offset the beneficial effects of thiazide. This cohort study investigates whether increased fracture risk due to thiazide-associated hyponatremia (TAH) outweighs the fracture-protective effects of thiazide.

## Materials and methods

### Data sources

This observational cohort study used data from Taiwan's National Health Insurance Research Database (NHIRD), which contains the healthcare data of more than 99% of Taiwan's population—more than 23 million people. Information recorded in the NHIRD includes date of birth, sex, diagnostic codes, drug prescriptions, and medical procedures. Medical claims reviews are performed quarterly to ensure the accuracy of the claim files.

For the present study, we retrieved data from the Longitudinal Health Insurance Database (LHID), a representative subset of NHIRD. This database includes groups—LHID2000, LHID2005, and LHID2010—of randomly sampled patients who were National Health Insurance beneficiaries in 2000, 2005, and 2010, respectively. Each group contains 1 million patients. There are no significant differences in age, sex, birth year, and average insured payroll-related amount between the sampled patients and the larger NHIRD database. We used LHID data from January 1997 to June 2013. This study was approved by the research ethics board of Kaohsiung Medical University Hospital (KMUHIRB-EXEMPT(II)-20160060).

### Study cohort

Patients were chosen for the study who had received a new prescription for thiazide lasting more than 28 days between January 1, 1998, and June 30, 2010. Among these patients, those diagnosed with hyponatremia (ICD-9-CM codes 276.1) within three years after initiation of thiazide and continued thiazide prescription were selected for the TAH group. A control group was selected from thiazide users without hyponatremia. Each TAH group patient was matched with four control patients based on age and sex. The index date for the TAH group was the date of prescription of thiazide after the occurrence of hyponatremia, while the index date for the control group was three years after initiation of thiazide. The index dates were chosen to ensure that the TAH group was a large enough sample size, and that there was enough time for the BMD-increasing effect of thiazide to develop. All enrolled study subjects were followed up from the index date till the appearance of a fracture, death, or the end of a 3-year period (whichever date came first). Fracture diagnoses were based on the use of corresponding ICD-9-CM codes (S1 Table) during emergency treatment or hospitalization. Fractures at the hip, vertebra, upper limb, and lower limb were emphasized in the analysis. Patients who were diagnosed with a fracture before the index date, died within 90 days after the index date, used a nasogastric tube, or suffered from adrenal insufficiency or hypothyroidism were excluded.

### Comorbidities and medication use

The comorbidities that were considered in this study included diabetes mellitus, hypertension, heart failure, chronic kidney disease, liver cirrhosis, stroke, osteoporosis, and peripheral artery disease (S1 Table). A comorbidity was included when the diagnosis code was used at least twice 1 year before the index date (outpatient), or at least once 1 year before the index date (inpatient). Charlson Comorbidity Index was used to evaluate the potential additive effects of multi-morbidity [25]. Co-administration of drugs, such as selective serotonin reuptake inhibitors (SSRIs), nonsteroidal anti-inflammatory drugs (NSAIDs), loop diuretics, potassium-sparing diuretics, and anti-osteoporotic agents, were included when the drug was prescribed more than 28 days before the index date.

### Sensitivity analysis

Several sensitivity analyses were performed to assess the robustness of the main findings under different assumptions, such as excluding comorbidities that may cause hyponatremia or falls, adjusting the index date, and modifying the outcome definition. The main analysis contained all TAH cases and controls. Sensitivity analyses included the following:

1. TAH patients enrolled in the main analysis and controls excluding history of chronic kidney disease, liver cirrhosis, and heart failure.
2. TAH patients enrolled in the main analysis and controls excluding history of chronic kidney disease, liver cirrhosis, heart failure, peripheral artery disease, osteoporosis, and stroke.
3. Index date for TAH group as three years after the initiation of thiazide.
4. Outcome as ICD-9-CM code from emergency room only.
5. Outcome as ICD-9-CM code from hospitalization only.
6. Subgroup analysis stratified by baseline characteristics.

### Statistical analysis

Normally distributed continuous data were expressed as means ± standard deviations. Numerical data with non-normal distributions were expressed as medians and interquartile ranges. All data were expressed as frequency (percentage) or as means ± standard deviations. The Student’s *t*-test and Mann-Whitney U test were used to compare parametric continuous data between groups, whereas the chi-square test was used for categorical data. The differences between the proportions of individuals who experienced a fracture in the TAH group and control groups during the 3-year follow-up period were analyzed using the Kaplan-Meier method with a log-rank test. After the assumptions of proportional hazards were confirmed using Schoenfeld residuals trend tests, which examined the interactions between predictors and event time, the Cox proportional hazard model was applied to examine the association of hyponatremia with fracture. Additionally, a multivariable Cox proportional hazard regression was performed to analyze independent risk factors for fracture. Hazard ratios (HRs) and 95% confidence intervals (CIs) were determined. Statistical significance was inferred as a two-sided p < 0.05. Analyses were performed using the SAS statistical package (version 9·2; SAS Institute Inc., www.sas.com).

## Results

### Demographics of the study subjects

**Fig 1:**
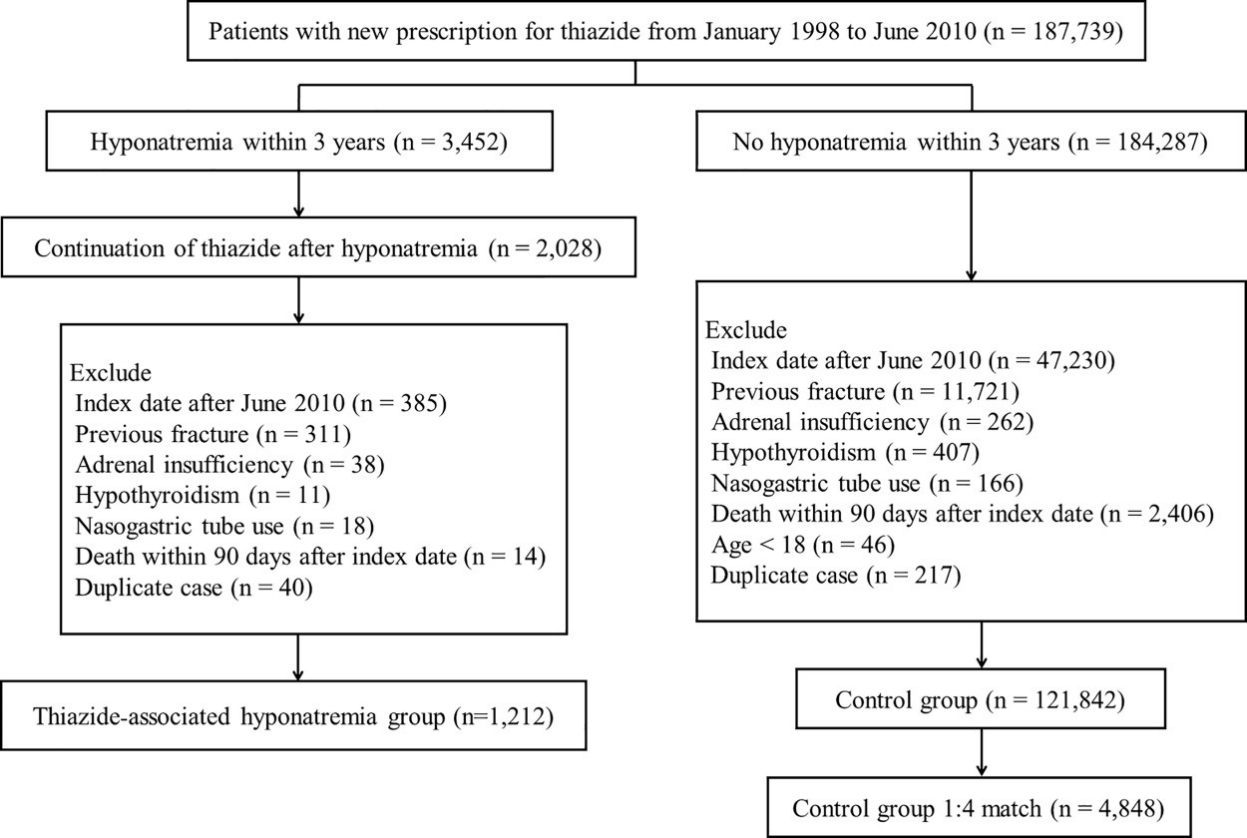
Study flowchart.

The study flow chart is shown in Fig 1. A total of 1212 patients were included in the TAH group; this number excluded individuals with an index date after June 30, 2010 (N = 385), with a fracture occurring before the index date (N = 311), with adrenal insufficiency (N = 38), with hypothyroidism (N = 11), with nasogastric tube use (N = 18), who died within 90 days after index date (N = 14), and duplicate cases (N = 40). Patient characteristics, comorbidity, and selected drug use are listed in Table 1. The mean age of the study population was 72.1 ± 11.0 years. Women comprised 61.5% of the study population. The mean follow-up period for the TAH group was 2.7 ± 0.7 years; for the control group, it was 2.9 ± 0.5 years. The patients in the TAH group had more comorbidities than those in the control group, and selected drug use was more common in the TAH group except for anti-osteoporotic medication. In the TAH group, the mean time from thiazide initiation to occurrence of hyponatremia was 1.22 ± 0.90 years, and the mean time from occurrence of hyponatremia to resuming thiazide was 4.8 ± 7.2 months. In the TAH group, symptomatic hyponatremia that required an emergency room visit or hospitalization recurred at least once in 288 patients (23.8%) during the follow-up period.

**Table 1:** Baseline characteristics of study subjects.

	TAH group (N = 1,212)	Control group (N = 4,848)	*P* value
	N (%)	N (%)	
Age (years)			0.9
< 60	161 (13.3%)	644 (13.3%)	
≥ 60	1051 (86.7%)	4204 (86.7%)	
Gender			
Male	467 (38.5%)	1868 (38.5%)	0.9
Female	745 (61.5%)	2980 (61.5%)	0.9
Comorbidity*			
Diabetes mellitus	534 (44.1%)	1206 (24.9%)	<0.001
Hypertension	1090 (89.9%)	3703 (76.4%)	<0.001
Heart failure	235 (19.4%)	448 (9.2%)	<0.001
Chronic kidney disease	84 (6.9%)	145 (3.0%)	<0.001
Liver cirrhosis	110 (9.1%)	267 (5.5%)	<0.001
Stroke	396 (32.7%)	641 (13.2%)	<0.001
Osteoporosis	92 (7.6%)	233 (4.8%)	<0.001
Peripheral artery disease	60 (5.0%)	114 (2.4%)	<0.001
Charlson comorbidity index	2 (1, 4)	1 (0, 2)	<0.001
Medication			
SSRIs	67 (5.5%)	155 (3.2%)	<0.001
NSAIDs	639 (52.7%)	3202 (66.0%)	<0.001
Potassium-sparing diuretics	334 (27.6%)	1164 (24.0%)	0.01
Loop diuretics	250 (20.6%)	779 (16.1%)	<0.001
Anti-osteoporotic medications**	38 (3.1%)	125 (2.6%)	0.33
Follow-up year	2.7 ± 0.7	2.9 ± 0.5	<0.001

TAH = thiazide-associated hyponatremia; SSRIs = selective serotonin reuptake inhibitors; NSAIDs = nonsteroidal anti-inflammatory drugs

*Comorbidities were included when the diagnosis code was used at least twice 1 year before the index date (outpatient), or at least once 1 year before the index date (inpatient).

**Anti-osteoporotic medications include bisphosphonate (ibandronic acid, zoledronic acid, alendronate, pamidronate, clodronate), calcitonin, raloxifene, teriparatide, RANKL inhibitor.

### Fracture risk among TAH patients compared to patients without hyponatremia

**Fig 2:**
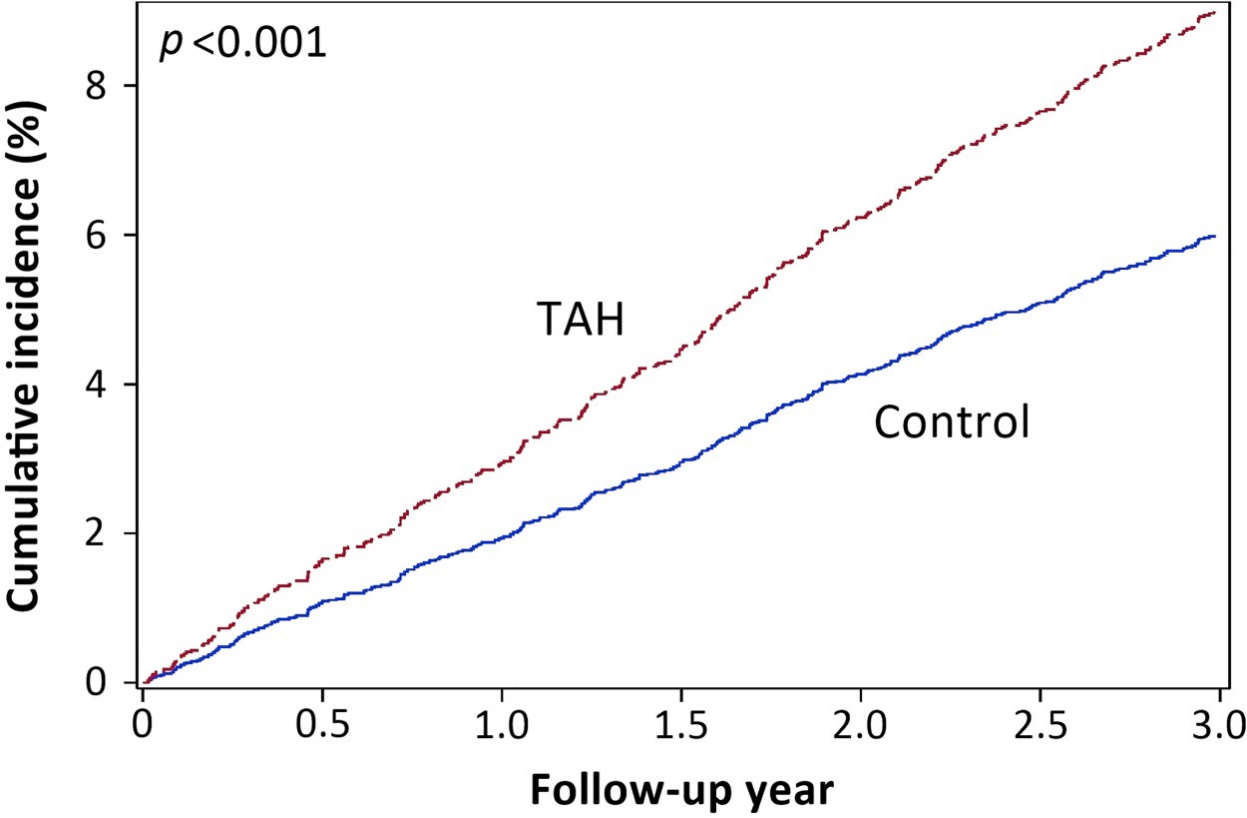
Cumulative incidence of fracture events using Kaplan–Meier estimator. Thiazide-assssociated hyponatremia (TAH) patients had increased fracture risk compared to matched comparison patients (log rank *p* < 0.001).

During the 3-year follow-up period, 103 fractures occurred in the TAH group (8.5%), equal to an incidence rate of 31.4 per 1000 person-years. In the control group, 286 fractures occurred (5.9%), equal to an incidence rate of 20.6 per 1000 person-years. (Table 2). The total fracture risk was higher in the TAH group than in the control group, with an incidence rate ratio of 1.44 (95% CI = 1.15–1.80, *P* = 0.001). The risk for both vertebra and hip fracture were higher in the TAH group than in the control group, with an incidence rate ratio of 1.96 (95% CI = 1.25–3.09, *P* = 0.003), and 1.62 (95% CI = 1.13–2.31, *P* = 0.01), respectively. The cumulative incidence of total fractures was higher in the TAH group than in the control group (*P* < 0.001), as determined by the Kaplan-Meier approach (Fig 2).

**Table 2:** Incident rate of fracture among thiazide-associated hyponatremia patients and control patients.

	TAH group (N = 1,212)		Control group (N = 4,848)		Incidence rate ratio	*P* value
	N	Incidence rate*	N	Incidence rate*		
Total fracture	103	31.43	286	20.62	1.44 (1.15–1.80)	0.001
Vertebra fracture	28	8.55	57	4.11	1.96 (1.25–3.09)	0.003
Hip fracture	42	12.82	104	7.50	1.62 (1.13–2.31)	0.01
Lower limb fracture (excluding hip fracture)	15	4.58	39	2.81	1.54 (0.85–2.79)	0.16
Upper limb fracture	17	5.19	89	6.42	0.76 (0.45–1.28)	0.31
Others	4	1.22	7	0.50	2.29 (0.67–7.81)	0.19

TAH = thiazide-associated hyponatremia

*Incidence of fracture: per 1,000 person-years

As shown in Table 3, the crude HR for total fractures in the TAH group was 1.52 (95% CI = 1.22–1.91, *P* < 0.001). Using multivariable Cox proportional hazards model analysis, the adjusted HR for total fractures was 1.47 (95% CI = 1.15–1.88, *P* = 0.002) after adjusting for age, gender, comorbidities (diabetes mellitus, hypertension, heart failure, chronic kidney disease, liver cirrhosis, stroke, osteoporosis, and peripheral artery disease), Charlson comorbidity index, and co-administration of medications (SSRIs, NSAIDs, potassium-sparing diuretics, loop diuretics, and anti-osteoporotic medications). The adjusted HR for vertebra fracture and hip fracture were 1.84 (95% CI = 1.12–3.01, *P* = 0.02) and 1.66 (95% CI = 1.12–2.46, *P* = 0.01), respectively. The risk of fracture in the upper limb and lower limb (hip fracture excluded) were not significantly different between the TAH group and the control group.

**Table 3:** Cox regression models for fracture risks of thiazide-associated hyponatremia patients compared to control patients.

	Model 1	*P* value	Model 2	*P* value	Model 3	*P* value
Total fracture	1.52 (1.22–1.91)	<0.001	1.35 (1.06–1.73)	0.02	1.47 (1.15–1.88)	0.002
Vertebra fracture	2.07 (1.32–3.26)	0.002	1.73 (1.06–2.83)	0.03	1.84 (1.12–3.01)	0.02
Hip fracture	1.71 (1.20–2.45)	0.003	1.49 (1.01–2.21)	0.04	1.66 (1.12–2.46)	0.01
Lower limb fracture (excluding hip fracture)	1.63 (0.90–2.96)	0.11	1.64 (0.85–3.17)	0.14	1.76 (0.91–3.42)	0.09
Upper limb fracture	0.81 (0.48–1.36)	0.42	0.73 (0.42–1.27)	0.26	0.77 (0.44–1.35)	0.37
Others	2.42 (0.71–8.27)	0.16	1.86 (0.49–7.10)	0.36	2.51 (0.66–9.57)	0.18

Model 1: Unadjusted model

Model 2: Adjusted for age, gender, and comorbidities (diabetes mellitus, hypertension, heart failure, chronic kidney disease, liver cirrhosis, stroke, osteoporosis, peripheral artery disease), and Charlson comorbidity index

Model 3: Adjusted for age, gender, comorbidities, Charlson comorbidity index, and medications (selective serotonin reuptake inhibitors, nonsteroidal anti-inflammatory drugs, potassium-sparing diuretics, loop diuretics, and anti-osteoporotic medications).

### Other risk factors associated with fracture among thiazide users

As shown in Table 4, multivariable Cox regression analysis demonstrated that being female was the leading risk factor for fracture (adjusted HR, 2.05, 95% CI = 1.61–2.61, *P* < 0.001), followed by use of NSAIDs (adjusted HR, 1.58, 95% CI = 1.26–1.99, *P* < 0.001), TAH, and age (adjusted HR, 1.03, 95% CI = 1.02–1.04, *P* < 0.001).

**Table 4:** Risk factors associated with fractures among all subjects by multivariable Cox regression analysis.

Variables	Multivariate analysis	
	HR (95% CI)	*P* value
Thiazide-associated hyponatremia	1.47 (1.15–1.88)	0.002
Age (per year)	1.03 (1.02–1.04)	<0.001
Female sex	2.05 (1.61–2.61)	<0.001
Diabetes mellitus	1.18 (0.92–1.52)	0.20
Hypertension	1.09 (0.84–1.42)	0.52
Heart failure	1.08 (0.79–1.48)	0.63
Chronic kidney disease	1.21 (0.73–2.03)	0.46
Liver cirrhosis	0.90 (0.57–1.42)	0.65
Stroke	1.14 (0.87–1.50)	0.34
Osteoporosis	0.81 (0.53–1.23)	0.33
Peripheral artery disease	0.79 (0.43–1.46)	0.46
Charlson comorbidity index	1.01 (0.93–1.10)	0.78
SSRIs	1.14 (0.70–1.86)	0.60
NSAIDs	1.58 (1.26–1.99)	<0.001
Potassium-sparing diuretics	0.91 (0.72–1.15)	0.42
Loop diuretics	1.21 (0.93–1.56)	0.16
Anti-osteoporotic medications	1.37 (0.83–2.26)	0.21

HR = hazard ratio; CI = confidence interval; SSRIs = selective serotonin reuptake inhibitor; NSAIDs = nonsteroidal anti-inflammatory drugs

### Sensitivity analysis

**Fig 3:**
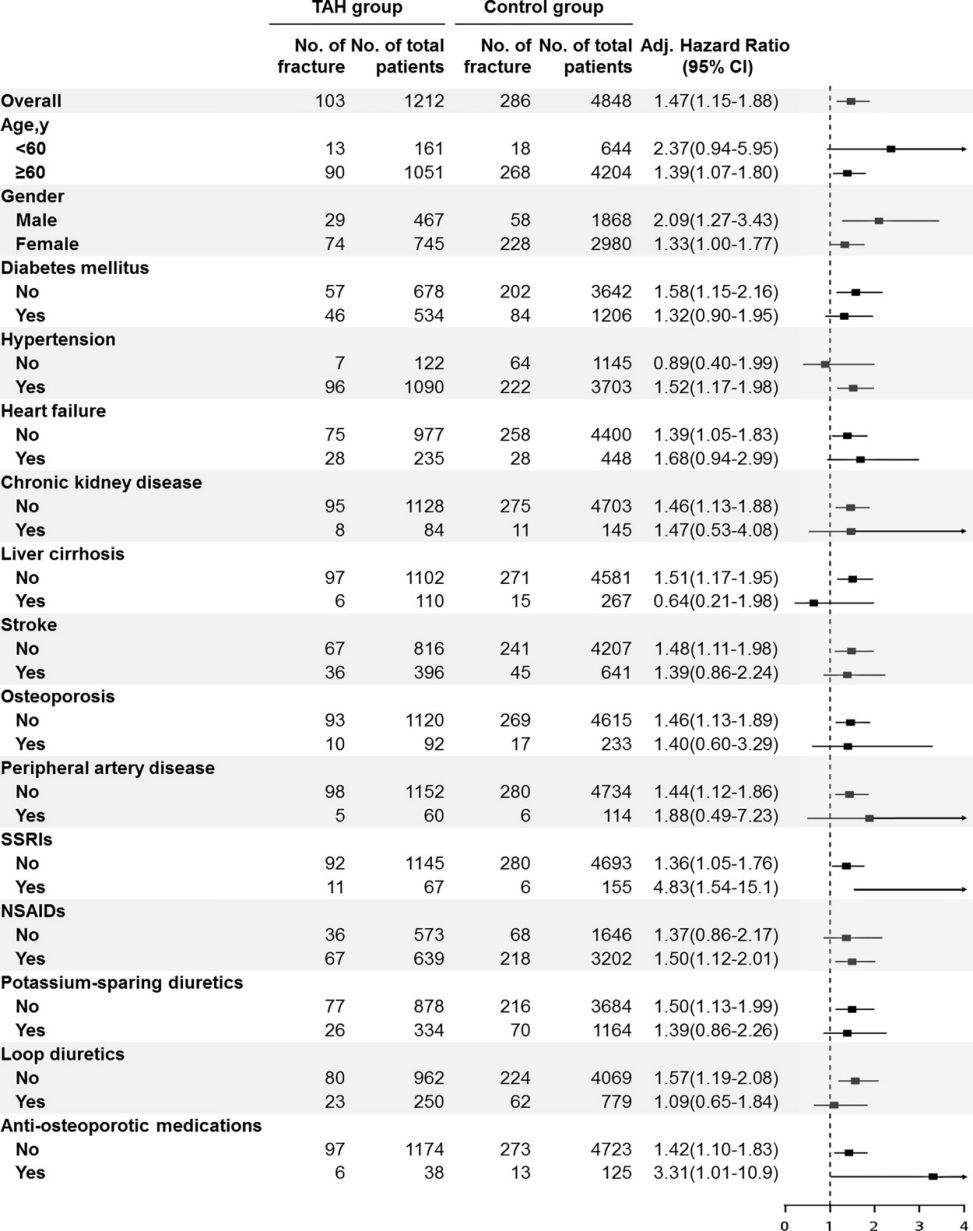
Subgroup analysis of association of thiazide-associated hyponatremia (TAH) with the risk of fracture.

Among all the subgroup analyses based on different assumptions, TAH group patients were consistently associated with significantly increased risk of fracture compared with control group patients (S2–S4 Tables). Using a multivariable Cox proportional hazards model, the adjusted HR for TAH group patients was 1.42 (95% CI, 1.06–1.91; *P* = 0.02) after excluding those with a history of chronic kidney disease, liver cirrhosis, and heart failure, 1.57 (95% CI, 1.09–2.26; *P* = 0.02) after excluding those with history of chronic kidney disease, liver cirrhosis, heart failure, peripheral artery disease, osteoporosis, and stroke, 1.52 (95% CI, 1.04–2.21; *P* = 0.03) after modifying the outcome to include ICD-9-CM codes from the emergency room only, and 1.41 (95% CI, 1.02–1.96; *P* = 0.04) after modifying the outcome to include ICD-9-CM codes from hospitalization only. The adjusted HR was 1.28 (95% CI, 0.97–1.69; *P* = 0.077) after moving the index date of the TAH group to 3 years after initiation of thiazide. Subgroup analysis also revealed similar trends (Fig 3).

## Discussion

In this population-based study, we found that thiazide users with hyponatremia have a higher risk of fracture than thiazide users without hyponatremia. This is important for physicians to know when evaluating the effects of thiazide medications. Once hyponatremia occurs, thiazide cannot guarantee decreased risk of fractures, meaning that the fracture risk associated with TAH may outweigh the fracture-protective effect of thiazide. We also demonstrated that patients who are female, older, and who use NSAIDs are at increased risk of fracture among thiazide users. Sensitivity test results validated the main findings that the risk of fracture was consistently higher in the TAH group than in the control group.

Chow et al. and De Vecchis et al. reported that TAH was associated with fracture in a univariate analysis but not in a multivariate analysis [26,27]. However, in both retrospective studies thiazide could have been stopped after a symptomatic hyponatremic episode; in such cases, the duration of hyponatremia would have been too short to increase fracture risk. In our study, patients in the TAH group continued thiazide use after the occurrence of hyponatremia, and nearly one-fourth of them had recurrent hyponatremia, which may indicate a longer duration of hyponatremia. Prolonged chronic hyponatremia has been associated with greatly increased hip fracture risk in the elderly population [22]. It is possible that the variation in the duration of hyponatremia among studies led to different results.

Arampatzis et al. conducted a case-control study and reported that hyponatremia during the use of furosemide was associated with an increased risk of fractures, but that hyponatremia with thiazide was not, in contrast to our study results [28]. However, that study did not take into account diseases that can cause hyponatremia, such as heart failure, liver cirrhosis, chronic kidney disease, hypothyroidism, and adrenal insufficiency. In this cross-sectional study, it is impossible to know whether the use of diuretics or the hyponatremia occurred first, which could lead to biases. In our study, we ensured that thiazide was being used prior to the incidence of hyponatremia, and comorbidities that may induce hyponatremia were considered and adjusted in the multivariable analysis.

In the present study, TAH increased the risk of fractures in both the hip and vertebra. The mechanisms for fracture in these two anatomical sites are very different. While 90% of hip fractures are the result of a fall [29], vertebral compression fractures are usually caused by osteoporosis [30]. Vertebral fractures in patients with severe osteoporosis may be caused by trauma that is relatively mild compared to a fall, such as vigorous sneezing, lifting a trivial object, or muscle contraction [31]. Based on this, our results indicate that TAH may induce fractures via two separate mechanisms, falls and osteoporosis. This is consistent with previous studies [32]. Several previous studies demonstrated that osteoporosis did not play an important part in the occurrence of fractures in patients with hyponatremia. Kinsella et al. reported that hyponatremia was associated with fracture occurrence independent of osteoporosis [33]. However, that study may have underestimated the role of osteoporosis in fracture by excluding patients with vertebral fractures. Hoorn et al. reported that hyponatremia was associated with increased risk of prevalent vertebral fractures and incident nonvertebral fracture but not with lower BMD [34]. However, serum sodium was only measured at baseline in that study, and thus it was not clear whether the hyponatremia was transient or chronic. The association of hyponatremia and osteoporosis has been demonstrated to be dose-dependent and time-dependent [35]. The longer-lasting and more severe the hyponatremia is, the higher the risk of developing osteoporosis. Thus, if hyponatremia is transient, it may not cause osteoporosis.

Osteoporosis, diagnosed by low BMD, is considered a risk factor for fracture [36]. Interestingly, in our multivariable analysis, osteoporosis was not statistically associated with increased fracture risk among thiazide users. It is possible that some patients with osteoporosis were not identified because not every study subject undertook screening by dual-energy X-ray absorptiometry, which is not reimbursed by Taiwan National Health Insurance. Osteoporosis could be underdiagnosed because it is usually asymptomatic until significant bone loss became evident and fractures occurred. Kung et al. reported that BMD measurement was only performed in 28.2% of patients prior to fragility fractures in Asian countries [37]. Because the “silent” osteoporosis patients could have been underestimated, the association between osteoporosis and fractures in our study should be interpreted with caution. Though most anti-osteoporosis medications were considered, drugs such as calcium and vitamin D supplements are available without a prescription, making them difficult to be taken into consideration in this study.

Consistent with previous studies, our results indicated that NSAID use is associated with increased risk of fracture [38]. Nonetheless, NSAID users have been reported to have higher BMD [39], possibly via the inhibition of prostaglandin synthesis, a known stimulator for bone resorption. Adverse effects on the central nervous system such as dizziness, headaches, mood alteration, and confusion have been reported among patients using NSAIDs [40]. NSAID exposure has been associated with increased risk of accidental falls [41]. It is possible that this increased risk of falls outweighs the benefits of increased BMD, causing increased fracture risk among NSAID users. However, confounding by indication cannot be totally excluded because patients taking NSAIDs could be more likely to have pain and therefore poor mobility, which may increase the risk of fracture.

In the present study, loop diuretics are not associated with increased fracture risk. Previous meta-analysis has conflicted with this result, concluding that loop diuretics were associated with increased risk of total fractures and hip fractures [42]. The strength of that meta-analysis may have been reduced by the varying levels of quality and high degree of heterogeneity among studies used. Dose and duration of loop diuretics have also been reported to influence the association between the use of loop diuretics and fracture risk. If most patients in a study received a high dose of loop diuretics, the results may show no effect or a decreased fracture risk, whereas if most patients received a low dose of loop diuretics, the results may reveal increased fracture risk [43].

The main strength of our study is that the results were based on an analysis of a large sample population, and the main finding was validated by several sensitivity analyses. However, our study has some limitations. First, this is a retrospective cohort study based on diagnostic codes and prescription history. Data regarding some important clinical features (such as body weight, height, strength, balance, physical activity, and blood pressure), laboratory parameters (such as serum sodium level and BMD), medication used by patients by themselves (such as calcium and vitamin D supply), and dietary patterns (salt content in diet and water intake) were not recorded in the NHIRD. Without these data, it is difficult to quantify the severity of hyponatremia. In addition, we cannot adjust for body mass index in the multivariable Cox regression analysis, although low body mass had been considered a risk factor for fracture in thiazide users [26]. Besides, we may underestimate the incidence of hyponatremia by analysis of databases using diagnostic codes, which were keyed-in manually by clinicians rather than generated automatically by the laboratory system. Nevertheless, when a diagnosis of hyponatremia was made, it was likely that hyponatremia indeed existed and was of clinical importance so that clinicians were motivated to enter the diagnostic code. That means the diagnosis of hyponatremia in the present study is more specific but may be less sensitive. Second, evidence deriving from any cohort study is generally considered to be of poorer methodological quality than data obtained from randomized trials, because cohort study design is associated with a higher likelihood of biases related to confounder adjustment. Since our study design was observational, a certain degree of residual confounding cannot be ruled out. For example, patients in the TAH group had more comorbidities and seemed more frail, which in itself could be associated with increased risk of fracture. Third, the age- and sex-matched and multivariable analysis were carried out to reduce potential confounding effects in the study. It is possible, but less likely, that residual confounding effects may still partially distort the result. Fourth, fracture diagnoses could not be verified through chart reviews, so it is possible that the study missed some patients with obscure fractures that caused few symptoms and could only be identified using radiographic studies. By analyzing clinically diagnosed fractures and not performing screening radiography, we might have underestimated the total number of radiographic fractures in both the TAH and control patients. However, these factors are not expected to have severely biased the results of this study because of the easy accessibility and high coverage of the universal health insurance program in Taiwan. Finally, because the study used data from Taiwan, most of the subjects are of Chinese ethnicity. It is uncertain whether the study findings can be generalized to other ethnic groups.

In conclusion, TAH patients have increased risk of fracture events compared to non-TAH patients. Our study has important clinical implications—though thiazide has beneficial effects besides lowering blood pressure, possible adverse effects should be noted. Because TAH may occur rapidly, serum sodium levels should be closely monitored after thiazide initiation, especially in high-risk patients. Once hyponatremia occurs, the necessity of continuing thiazide should be reconsidered to strike a balance between risk and benefit.

## Supporting information

S1 Table The corresponding ICD-9-CM codes for the diagnoses of diseases examined in this study. (DOCX)

S2 Table Sensitivity analyses–comorbidity. (DOCX)

S3 Table Sensitivity analyses–index date. (DOCX)

S4 Table Sensitivity analyses–outcome. (DOCX)
