# Supplementary material for: Thiazide-associated hyponatremia attenuates the fracture-protective effect of thiazide: A population-based study
Source: PLoS One. 2018 Dec 7;13(12):e0208712. doi: 10.1371/journal.pone.0208712 (PMC6285977; doi:10.1371/journal.pone.0208712)
Supplement: S1 Table — (DOCX) [file pone.0208712.s001.docx]

S1 Table. The corresponding ICD-9-CM codes for the diagnoses of diseases examined in this study

| Disease | Corresponding ICD-9-CM codes |
| --- | --- |
| Hyponatremia | 276.1 |
| Comorbidity |  |
| Diabetes mellitus | 250 |
| Hypertension | 401–405 |
| Heart failure | 398.91, 402.01, 402.11, 402.91, 404.01, 404.03, 404.11, 404.13, 404.91, 404.93, 428 |
| Chronic kidney disease | 585 |
| Liver cirrhosis | 571 |
| Stroke | 430-438 |
| Osteoporosis | 733.0, 733.01, 733.02, 733.03, 733.09 |
| Peripheral artery disease | 440.0, 440.2, 440.3, 440.8, 440.9, 443, 444.0, 444.22, 444.8, 447.8, 447.9 |
| Adrenal insufficiency | 255.4, 255.5 |
| Hypothyroidism | 244.9 |
| Outcome |  |
| Fracture | 805, 806, 808, 810, 811, 812, 813, 814, 815, 816, 817, 820, 821, 822, 823, 824, 825, 826 |
| Vertebra fracture | 805 |
| Hip fracture | 820 |
| Lower limb fracture | 820, 821, 822, 823, 824, 825, 826 |
| Upper limb fracture | 810, 811, 812, 813, 814, 815, 816, 817 |
