# Supplementary material for: Thiazide-associated hyponatremia attenuates the fracture-protective effect of thiazide: A population-based study
Source: PLoS One. 2018 Dec 7;13(12):e0208712. doi: 10.1371/journal.pone.0208712 (PMC6285977; doi:10.1371/journal.pone.0208712)
Supplement: S4 Table — (DOCX) [file pone.0208712.s004.docx]

S4 Table. Sensitivity analyses – outcome

|  | TAH group | | Control group | |  | |
| --- | --- | --- | --- | --- | --- | --- |
|  | No. of total fracture | No. of patients | No. of total fracture | No. of patients | Adjusted HR  (95% CI) | *P* value |
| Main analysis  (outcome as ICD-9-CM code from emergency room or hospitalization) | 103 | 1,212 | 286 | 4,848 | 1.47 (1.15 - 1.88) | 0.002 |
| Outcome as ICD-9-CM code from emergency room only | 44 | 1,212 | 121 | 4,848 | 1.52 (1.04 - 2.21) | 0.03 |
| Outcome as ICD-9-CM code from hospitalization only | 59 | 1,212 | 165 | 4,848 | 1.41 (1.02 - 1.96) | 0.04 |

TAH = thiazide-associated hyponatremia; HR = hazard ratio; CI = confidence interval

Adjusted for age, gender, comorbidities (diabetes mellitus, hypertension, heart failure, chronic kidney disease, liver cirrhosis, stroke, osteoporosis, peripheral artery disease), Charlson comorbidity index, and medications (selective serotonin reuptake inhibitors, nonsteroidal anti-inflammatory drugs, potassium-sparing diuretics, loop diuretics, and anti-osteoporotic medications).
